# Elevated expression of hyaluronan synthase 2 associates with decreased survival in diffusely infiltrating astrocytomas

**DOI:** 10.1186/s12885-018-4569-1

**Published:** 2018-06-18

**Authors:** Mari Valkonen, Hannu Haapasalo, Kirsi Rilla, Kristiina Tyynelä-Korhonen, Ylermi Soini, Sanna Pasonen-Seppänen

**Affiliations:** 1 Institute of Biomedicine, University of Eastern Finland, 70211 Kuopio, Finland; 2 Department of Pathology, University of Tampere and Fimlab Laboratories, Tampere, Finland; 3 Cancer Center, Kuopio University Hospital, Kuopio, Finland; 4 Institute of Clinical Medicine/ Clinical Pathology, University of Eastern Finland, Kuopio, Finland; 5 Department of Clinical Pathology, Kuopio University Hospital, Kuopio, Finland; 6 Cancer Center of Eastern Finland, Kuopio, Finland

**Keywords:** Hyaluronan synthase, Astrocytoma, Prognosis, Glioma, Hyaluronan

## Abstract

**Background:**

Diffusely infiltrating astrocytomas originate from astrocytic glial cells or their precursor cells and are the most common type of brain tumors in adults. In this retrospective study, we investigated the content of hyaluronan, its cell surface receptor, CD44 and the expression of hyaluronan metabolizing enzymes, in these aggressive tumors. Hyaluronan is the main component of extracellular matrix in the brain. In many tumors, aberrant hyaluronan metabolism implicates aggressive disease progression and metastatic potential.

**Methods:**

Our material consisted of 163 diffusely infiltrating astrocytomas (WHO grades II-IV). Tumor samples were processed into tissue microarray (TMA) blocks. The TMA sections were stained for hyaluronan, CD44, hyaluronan synthases 1–3 (HAS1–3) and hyaluronidase 2 (HYAL2). The immunostaining results were compared with χ2 –test or with Kruskal-Wallis test for correlation with clinicopathological parameters and survival analyses were done with Kaplan-Meier log rank test and Cox regression.

**Results:**

Hyaluronan and CD44 were strongly expressed in astrocytic gliomas but their expression did not correlate with WHO grade or any other clinicopathological parameters whereas high HAS2 staining intensity was observed in *IDH1* negative tumors (*p* = 0.003). In addition, in non-parametric tests increased HAS2 staining intensity correlated with increased cell proliferation (*p* = 0.013) and in log rank test with decreased overall survival of patients (*p* = 0.001). In the Cox regression analysis HAS2 expression turned out to be a significant independent prognostic factor (*p* = 0.008).

**Conclusions:**

This study indicates that elevated expression of HAS2 is associated with glioma progression and suggests that HAS2 has a prognostic significance in diffusely infiltrating astrocytomas.

**Electronic supplementary material:**

The online version of this article (10.1186/s12885-018-4569-1) contains supplementary material, which is available to authorized users.

## Background

Hyaluronan is a large glycosaminoglycan composed of repetitive units of N-acetylglucosamine and glucuronic acid. In mammalian cells, there are three hyaluronan synthases (HAS1, HAS2 and HAS3) which produce various sizes of hyaluronan in the inner leaflet of the plasma membrane. During its synthesis, hyaluronan is directly extruded through the plasma membrane to the cell surface or into the extracellular matrix [1]. Connective tissues contain the largest amounts of hyaluronan, but it is synthesized abundantly also in other tissues, including central nervous system [2]. In vitro*,* hyaluronan increases migration of astrocytes which depends on hyaluronan receptor CD44 [3]. During brain development hyaluronan has an essential role in the migration of neural cells [4]. In many cancers, alterations in hyaluronan content and metabolism affect progression and prognosis of disease [5–8]. In breast cancer, high levels of HAS1–3 in stromal cells associate with decreased survival [9]. Silencing of HAS2 correlates with increased expression of tissue metalloproteinase inhibitor 1 (TIMP-1) leading into decreased invasive capability in breast cancer cells [10].

Gliomas originate from different types of neuroglial cells or their precursors. Diffusely infiltrating astrocytomas are the most common type of gliomas. They are divided into three categories (grades II-IV) by WHO [11, 12]. Human glioma cell lines produce hyaluronan and CD44, which are essential for adhesion, invasion and migration of glioma cells [13–15]. It has been proposed that aggressiveness of glioblastoma cell lines depends on the co-expression of HASes and hyaluronidases [16]. Moreover, it has been reported that in high grade astrocytomas (grades III-IV) the expression of CD44 is elevated [14]. Knockdown of CD44 in glioblastoma cell lines decreases tumor growth and sensitizes them to cytotoxic drugs [17].

Although the functions of hyaluronan and CD44 have been previously investigated in gliomas in vitro, the roles of HASes and HYALs are unclear. Here we studied the expression of hyaluronan related proteins in diffusely infiltrating astrocytomas, and compared the results with histopathological and clinical parameters.

## Methods

This retrospective study consisted of 163 WHO grade II-IV diffusely infiltrating astrocytomas. Clinical data was obtained from 150 patients (Table 1). Samples without clinical information were excluded from the statistical analyses. Samples were obtained with maximal safe resection from surgically operated patients at the Tampere University Hospital, Tampere, Finland, during 1983–2001. Data on radio- and chemotherapy was known in 105 and 101 patients, respectively. Of these, 25 received only radiotherapy, whereas 16 received only chemotherapy. Forty-three patients received both radio- and chemotherapy. Forty-six patients received also Temozolomide treatment for progressive astrocytoma. Follow-up time ranged from 0.1 to 83.4 months (mean follow-up 18.0 months) (Table 1).

**Table 1:** Clinicopathological characteristics of the diffusely infiltrating astrocytoma patients (*n* = 150) according to WHO classification

Variable	Grade II	Grade III	Grade IV	Total
Number of cases	25 (16.7%)	6 (4.0%)	119 (79.3%)	150 (100.0%)
Gender				
Male	20 (80.0%)	1 (16.7%)	66 (55.5%)	87 (58.0%)
Female	5 (20.0%)	5 (83.3%)	53 (44.5%)	63 (42.0%)
Mean age at time of diagnosis (years)	40.6	46.2	58.3	54.8
Mean survival time (months)	36.5	29.5	13.4	18.0
Alive				
Yes	9 (36.0%)	2 (33.3%)	6 (5.0%)	17 (11.3%)
No	16 (64.0%)	3 (50.0%)	108 (90.8%)	127 (84.7%)
No information	0 (0.0%)	1 (16.7%)	5 (4.2%)	6 (4.0%)
Cause of death				
Glioma	16 (100.0%)	3 (100.0%)	108 (100.0%)	127 (100.0%)
Other causes	0 (0.0%)	0 (0.0%)	0 (0.0%)	
Surgical operation				
No	0 (0.0%)	0 (0.0%)	0 (0.0%)	0 (0.0%)
Resection	21 (84.0%)	6 (100.0%)	114 (95.8%)	141 (94.0%)
Biopsy	4 (16.0%)	0 (0.0%)	4 (3.4%)	8 (5.3%)
No information	0 (0.0%)	0 (0.0%)	1 (0.8%)	1 (0.7%)

The final study material included 25 grade II diffuse astrocytomas, 6 grade III anaplastic astrocytomas and 119 grade IV glioblastomas (including 3 gliosarcomas). Samples included both primary and recurred astrocytomas. Histopathological analyses and grading were performed in Fimlab Laboratories at Tampere University Hospital. Proliferation was analysed with Ki-67 staining (MIB1), *isocitrate dehydrogenase 1 (IDH1)* mutation with R132H point mutation-specific mouse monoclonal antibody (Dianova GmbH, Hamburg, Germany), EGFR amplification with chromogenic in situ hybridisation (CISH) and p53 status with immunohistochemistry (antibody: DO-7, Novocastra Laboratories, Newcastle, UK) as described previously [18, 19]. The histopathological stainings were analysed by experienced pathologists in Fimlab Laboratories at Tampere University Hospital. *IDH1* status was evaluated by whether there were IDH1-R132H -positive tumor cells in astrocytomas (Additional file 1: Figure S1). Brain tumor samples were formalin-fixed, paraffin-embedded and processed into tissue microarray blocks (TMA) [19]. The study protocol was approved by Ethical Board of Northern Savo Hospital District 108/2010, Ethical Committee of Tampere University Hospital, National Authority for Medicolegal Affairs of Finland and VALVIRA 9121/2010. The material also included two normal human cerebral brain tissue specimens from the Kuopio University Hospital obtained from areas adjacent to tumor tissue.

### Hyaluronan and CD44 stainings

Hyaluronan staining was performed similarly as in our previous work using a biotinylated hyaluronan-binding complex (bHABC) [8]. The specificity of the bHABC staining was controlled by *Streptomyces* hyaluronidase (data not shown). The staining for CD44 was done with Hermes3 antibody (a kind gift from Dr. Sirpa Jalkanen, University of Turku, Finland) as previously described [8].

### HAS1–3 and HYAL2 stainings

To detect HAS1–3 and hyaluronidase 2 in astrocytomas, specimens were incubated with goat polyclonal antibodies for HAS1–3 diluted in 1% BSA (HAS1 antibody 1:100, HAS2 antibody 1:120 and HAS3 antibody 1:80 dilution, Santa Cruz Biotechnology, Santa Cruz, CA) or with rabbit polyclonal antibody for HYAL2 (1100 Abcam, Cambridge, UK). The stainings were performed as described previously [8]. Tissue sections without the primary antibodies were used as negative controls. The specificity of HAS2 antibody was tested with peptide-blocking (sc-34,067 P for HAS2, Santa Cruz Biotechnology) in the glioma samples (Additional file 2: Figure S2).

### Evaluation of stainings

Intensity of stained TMA samples was estimated with a four-level scoring system (0 = negative, 1 = weak, 2 = moderate and 3 = strong staining intensity). Scoring of slides was performed independently by two researchers. The coverage of the stained tumor cells was evaluated as follows: 0 = under 5% (considered negative), 1 = 6–25%, 2 = 26–75% and 3 = over 76% of cells stained. Staining coverage and intensity results were combined creating a new variable, the staining INDEX. The INDEX-values were computed as follows: The staining intensity was multiplied with the coverage of the stained cells, thus the INDEX-values ranged from 0 to 9.

### Statistical analyses

Statistical analyses were done with SPSS Statistics (IBM SPSS Statistics 21.0) in Tampere University Hospital. Association between immunostainings and histopathological parameters were analysed with χ2 and Kruskal-Wallis test. Survival analyses were performed with Kaplan-Meier log rank test and Cox regression model. *P*-values < 0.05 were considered statistically significant.

## Results

Hyaluronan and CD44 are mainly localized in white matter in normal brain tissue.

**Fig. 1:**
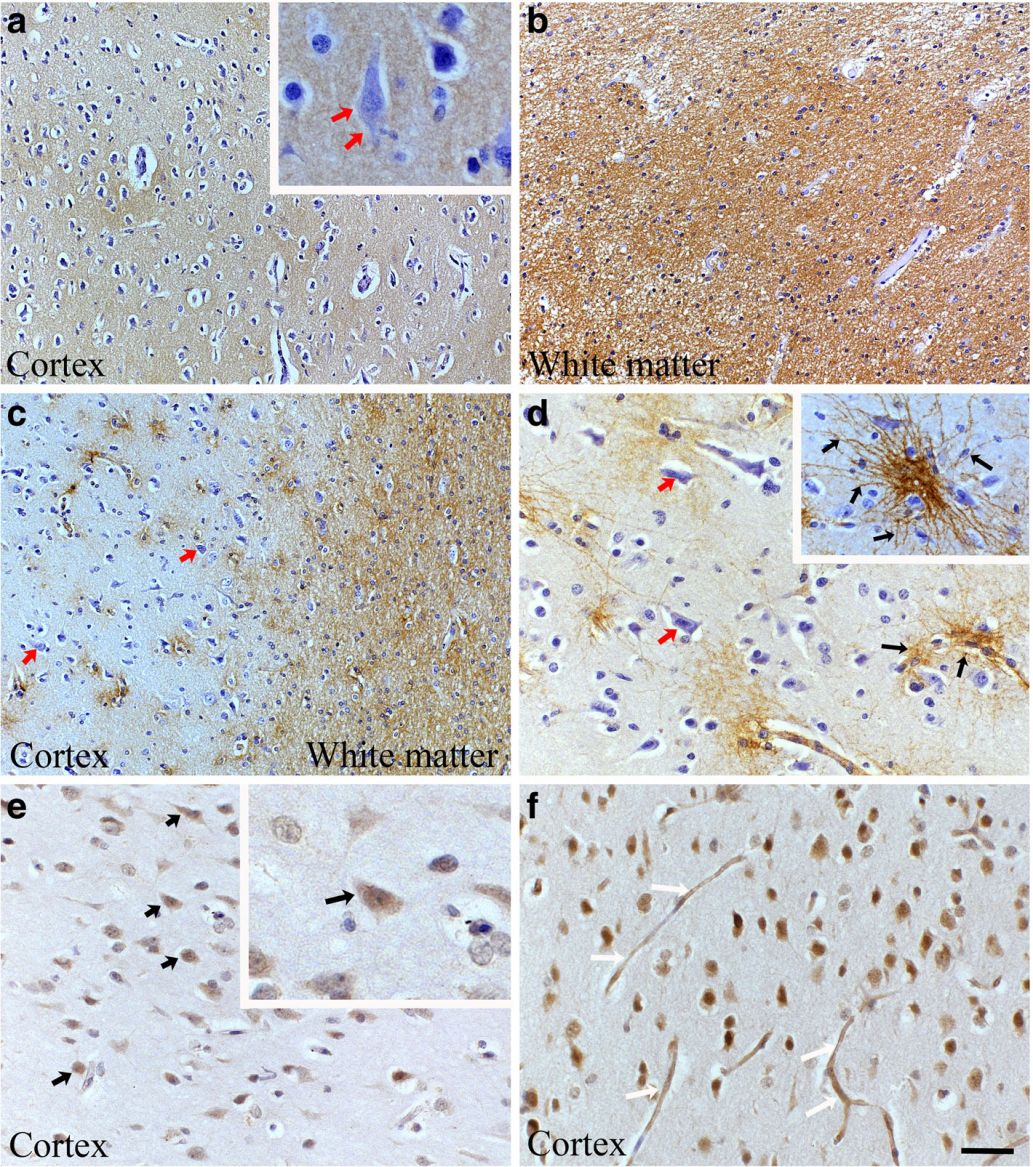
Hyaluronan, CD44, HAS2 and HYAL2 stainings in brain tissue samples adjacent to tumor areas. Hyaluronan (**a** and **b**) staining intensity is higher in white matter (**b**) than in the cortex (**a**). Likewise, CD44 (**c** and **d**) staining intensity is stronger in white matter than in the cortical areas (**c**). However, there is noticeable CD44 staining in perivascular areas (**d**, **d** insert), where astrocytes show strong CD44 immunoreactivity. HAS2 staining is generally weak (**e**), but there are some strongly HAS2 positive neurons and glial cells (black arrows, **e** insert). HYAL2 staining is more prominent in the cortical areas whereas white matter is HYAL2 negative (**f**). Endothelial cells (white arrows) express strongly HYAL2 (**f**). Scale bar in F is 50 μm

Cerebral brain tissue samples adjacent to tumor areas with some visible gliosis showed abundant staining for hyaluronan and CD44 (Fig. 1a, c). Both were mainly localized in the white matter (Fig. 1b, c). Neurons in the cortical areas were mostly negative or showed weak staining for hyaluronan and CD44 (Fig. 1a, c, and d insert, red *arrows*). CD44 localized strongly around perivascular areas, especially astrocytes showed intense CD44 immunopositivity (Fig. 1d, black *arrows*). In cortical brain, HAS2 expression was relatively low, but some of the glial cells and neurons were HAS2 –positive (Fig. 1e, black *arrows*). Interestingly, HYAL2 immunostaining was opposite to hyaluronan and CD44 (Fig.1f), localizing mainly in the cortical areas (Fig. 1f) whereas the white matter was negative for it. Glial cells were HYAL2 positive and in cortical capillaries, endothelial cells showed intense HYAL2 staining (Fig. 1f, white *arrows*).

### Hyaluronan and CD44 are abundantly stained in diffusely infiltrating astrocytomas

**Fig. 2:**
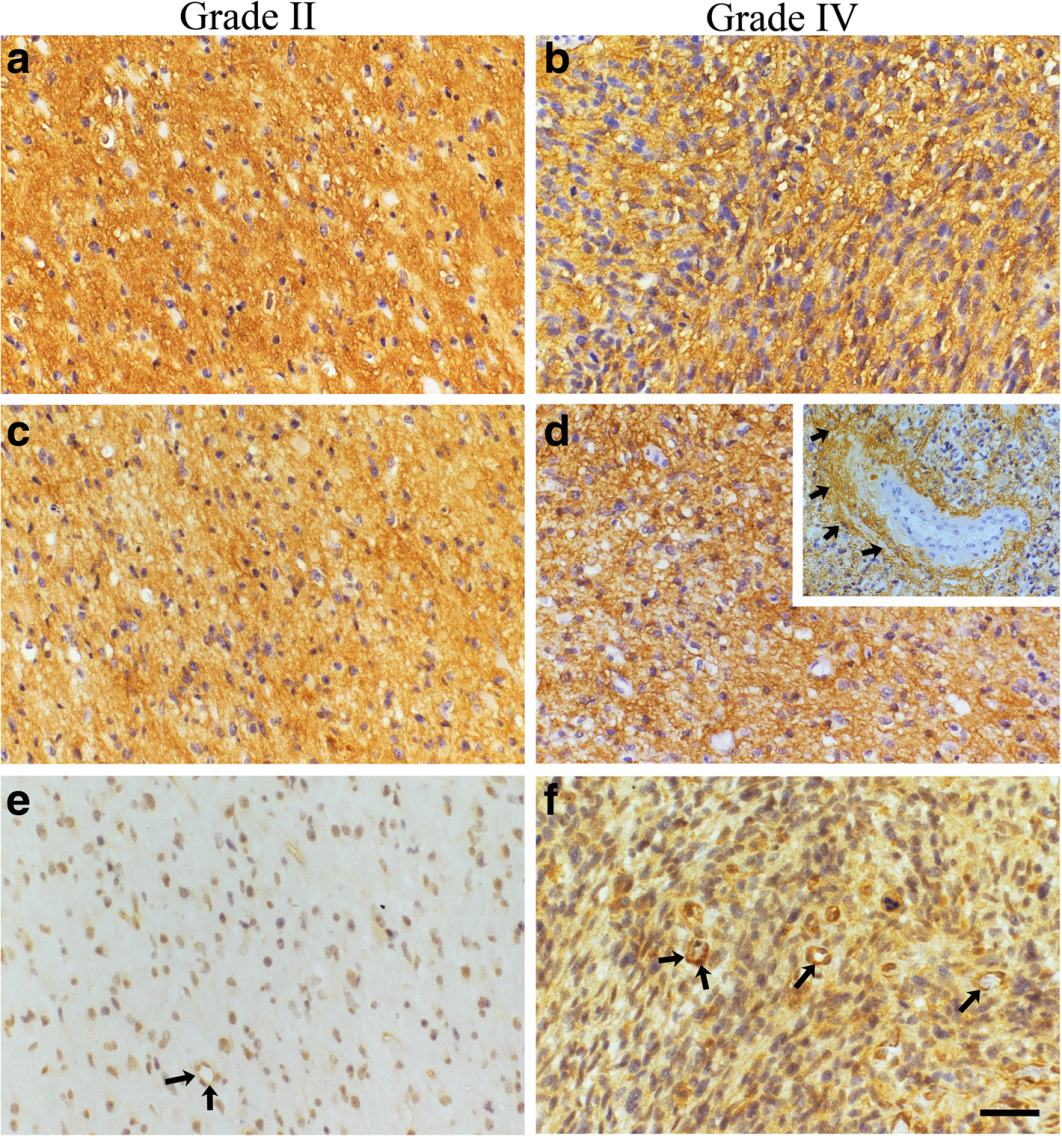
Hyaluronan, CD44 and HYAL2 stainings in grade II and grade IV astrocytomas. Hyaluronan (**a** and **b**) and CD44 (**c** and **d**) staining intensities are strong both in grade II (**a** and **c**) and grade IV (**b** and **d**) astrocytomas. CD44 is markedly expressed in the perivascular areas (**d**, insert, black arrows). HYAL2 staining intensity increases according to WHO grade (**e** and **f**). Endothelial cells show HYAL2 immunopositivity (**e** and **f**, black arrows). Scale bar in F is 50 μm

Hyaluronan and CD44 staining intensities were recorded from 136 and 120 specimens, respectively. Hyaluronan and CD44 showed intense membranous and cytoplasmic staining (Fig. 2a-d). 57% (77/136) and 42% (50/120) of the samples showed high hyaluronan and CD44 staining intensity respectively (Fig. 4a, b). Especially high CD44 staining was observed around vascular structures (Fig. 2d, insert). Hyaluronan or CD44 did not associate with WHO grade even when grade II-III tumors were combined and compared with grade IV tumors.

HYAL2 staining intensity was recorded from 133 (grade II = 21, grade III = 6 and grade IV = 106) samples. HYAL2 staining intensities were mostly weak (81/133) or moderate (29/133) (Fig. 2e, f and 4c). HYAL2 staining was mainly cytoplasmic, and it was also seen in endothelial cells (Fig. 2e, f). 25% (27/106) of grade IV tumors were moderately stained with HYAL2, whereas 7.4% (2/27) of grade II-III tumors had moderate HYAL2 staining (Fig. 4c). Increased HYAL2 staining intensities and increased HYAL2-INDEX values (*n* = 132) were associated with increased WHO grade (*p* = 0.049 and *p* = 0.001, respectively). When grade II-III tumors were combined and compared with grade IV tumors differences were not statistically significant.

### Elevated immunostaining intensities of HAS1–2 associate with increased WHO grade

**Fig. 3:**
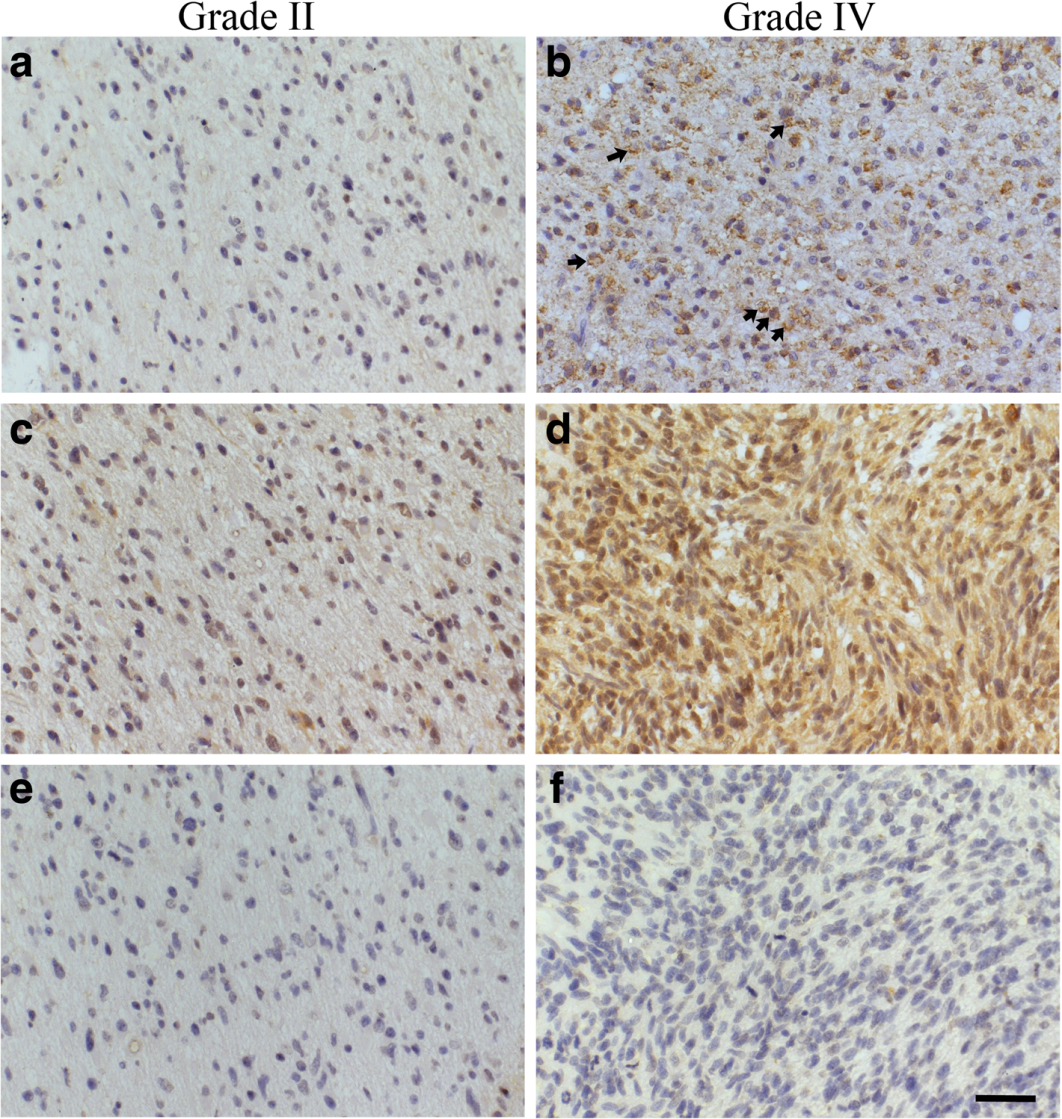
HAS1–3 immunostainings in grade II and grade IV astrocytomas. HAS1 staining intensity is higher in grade IV astrocytomas (**b**) than in grade II astrocytomas (**a**). Results are similar for HAS2, the staining intensity is higher in grade IV astrocytomas (**d**) than in grade II astrocytomas (**c**). HAS3 staining intensities are very weak or negative in grade II (**e**) and grade IV astrocytomas (**f**). Scale bar in F is 50 μm

**Fig. 4:**
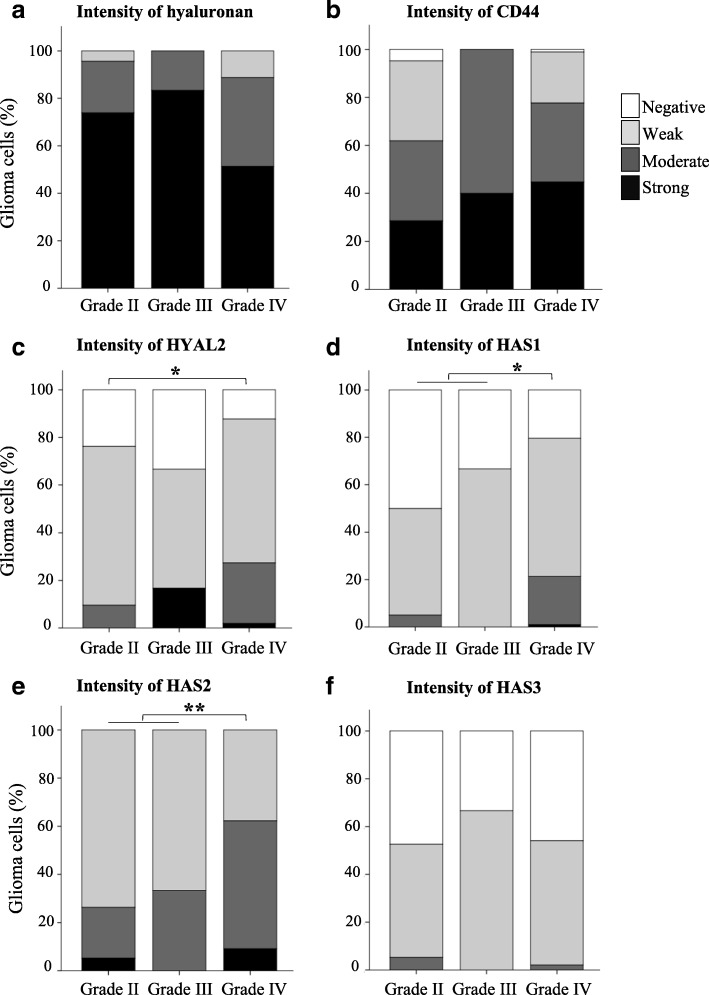
Hyaluronan, CD44, HYAL2, HAS1, HAS2 and HAS3 staining intensities in gliomas according to WHO grade. There are no statistically significant differences between the groups in hyaluronan (**a**) and CD44 (**b**) stainings. Intensity of HYAL2 staining increases according to WHO grade (**c**). HAS1 staining intensity is higher in grade IV astrocytomas compared to grade II and III astrocytomas (**d**). HAS2 staining intensity increases in grade IV astrocytomas compared to grade II-III astrocytomas (**e**). Most of the samples are HAS3 negative or the staining intensity is weak (**f**). * = *p*-value < 0.05, ** = *p*-value < 0.01

All HASes were expressed in diffusely infiltrating astrocytomas. HAS2 was the most prevalent isoform localizing diffusely in the cytoplasm and near the cell surface (Fig. 3c, d and 4e). HAS1 showed diffuse and granular staining pattern and it mainly localized in the cytoplasm but also occasional plasma membrane immunopositivity was observed (Fig. 3b). HAS1 staining intensity (*n* = 129) was not associated with WHO grade. However, grade IV tumors (*n* = 103) had increased HAS1 staining intensities compared to grade II-III tumors combined (*n* = 26). 46% (12/26) of grade II-III tumors were HAS1 negative and 50% (13/26) had weak HAS1 staining. 20% (21/103) of grade IV tumors were HAS1 negative, 58% (60/103) showed weak immunostaining and 20% (21/103) moderate staining intensity (*p* = 0.026, Fig. 4d).

HAS2 staining intensity was recorded from 123 specimens (grade II = 19, grade III = 6, grade IV = 98). Increased HAS2 immunostaining intensity was associated with higher WHO grade (*p* = 0.044, Fig. 4e). 53% (52/98) of grade IV tumors, 33% (2/6) of grade III tumors and 21% (4/19) of grade II tumors had moderate staining intensity. In other words, 72% (18/25) of grade II-III tumors were weakly stained for HAS2, whereas in grade IV tumors only 38% (37/98) were weakly stained (*p* = 0.009) (Fig. 4e). HAS2-INDEX was higher in grade IV tumors compared with grade II-III tumors combined (*p* = 0.015).

HAS3 immunostaining was the weakest of three HASes (Fig. 3e, f). Most of the samples were either negative (56/123) or weakly (64/123) HAS3 positive (*n* = 123) (Fig. 4f). There were no differences between the groups.

### *IDH1* mutation associates with low HAS2 immunostaining in diffusely infiltrating astrocytomas

**Fig. 5:**
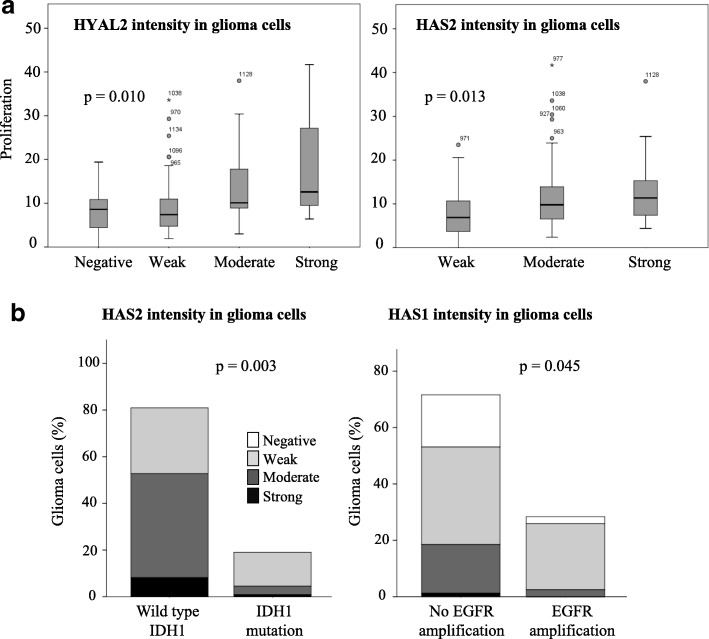
Associations with histopathological parameters. Increased intensities of HYAL2 and HAS2 associate statistically significantly with increased proliferation rates (MIB1, Ki-67 positive cells) (*p* = 0.010 and *p* = 0.013, respectively) (**a**). HAS1 staining intensity associates with EFGR amplification (**b**) and HAS2 staining intensity associates with *IDH1*-mutation (**b**)

In our material, *IDH1* mutation was associated with low HAS2 intensity (21 *IDH1*-mutation positive and 89 wild type *IDH1*) (*p* = 0.003, Fig. 5b). Furthermore, increased intensity of HAS2 and HYAL2 were associated with increased proliferation analysed with Ki-67 staining (*p* = 0.013 and *p* = 0.010, respectively, Fig. 5a).

HAS1 intensity was associated with EGFR amplification (*p* = 0.045, respectively, Chi-Square, Fig. 5b). EGFR amplification status was reported in 81 tumors with known HAS1 intensity value. EGFR amplification was found in 23 tumors – most of them showed weak HAS1 staining intensity (19/23) (Fig. 5b). Tumors without EGFR amplification had similar distribution of HAS1 intensity levels as in the whole material.

Hyaluronan, CD44 and HAS3 did not associate with any of the studied histopathological parameters (WHO grade, *IDH1,* p53, proliferation, EGFR).

### Increased HAS2 intensity associates with decreased overall survival

**Fig. 6:**
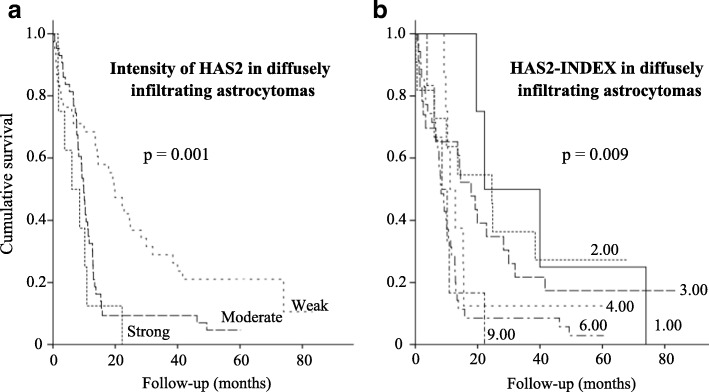
Patient survival according to HAS2 staining intensity. Elevated HAS2 intensity (**a**) and high HAS2-INDEX (**b**) correlate with decreased overall survival in diffusely infiltrating astrocytomas (*p* = 0.001, *p* = 0.009, Kaplan-Meier log-rank test)

In univariate analyses, higher HAS2 (*n* = 89) staining intensity was associated strongly with decreased overall survival time (*p* = 0.001, Fig. 6a), similarly as high HAS2-INDEX values (*n* = 87, *p* = 0.009, Fig. 6b).

Because HAS2 intensity was associated with overall survival in univariate analyses we performed multivariate analysis. Variables used were: WHO grade, *IDH1* mutation status, p53, EGFR amplification and HAS2 staining intensity. Sixty-two specimens with complete data were included in the analysis. In the Cox multivariate analysis, HAS2 staining intensity was the only significant independent factor (*p* = 0.008, Hazard Ratio = 1.685, 95% CI 1.144–2.481 for HAS2 staining intensity, other variables not significant in the final equation, *n* = 62, stepwise Cox regression, Additional file 3: Table S1).

## Discussion

Our results indicate that especially HAS2 and to a lesser extent HYAL2 and HAS1 are involved in the progression of diffusely infiltrating astrocytomas. The present work shows that increased immunostaining intensities of HAS1, HAS2 and HYAL2 associate with increased WHO grade of the tumors. Low HAS2 immunostaining was associated with *IDH1* mutation, a known positive prognostic factor in diffusely infiltrating astrocytomas. Increased intensities of HAS2 and HYAL2 were associated with increased tumor cell proliferation. Survival analyses indicated that elevated HAS2 staining intensity is associated with decreased overall survival of the patients and is an independent prognostic factor in infiltrating astrocytoma.

Hyaluronan and CD44 have been shown to promote glioma cell invasion, migration and adhesion [14, 15, 20]. In gliomas, the cleavage of CD44 is important for tumor cell migration, invasion and adhesion and this is induced by matrix metalloproteinase-9 (MMP-9) [21]. Hyaluronan, on the other hand, increases migration of glioma cells via up-regulation of osteopontin [20]. In our material both hyaluronan and CD44 were abundantly expressed in all WHO grade (II-IV) astrocytomas but there were no statistically significant differences between the grades. In this respect, our results are contradictory to a previous report where CD44 expression was increased in high grade compared to low grade astrocytomas [14]. Our material, however, had a higher number of cases. In general, high CD44 expression is linked to mesenchymal subtype of glioblastoma and this associates with poor prognosis [22]. Interestingly, our results show that hyaluronan and CD44 do not associate with prognosis or any tested histopathological parameters.

Our present data shows that increased HAS2 immunostaining intensity is a negative prognostic factor in astrocytomas associating significantly with decreased survival of patients both in univariate and multivariate tests. In breast cancer cells knockdown of HAS2 inhibits cell proliferation, migration and invasion, increases apoptosis and number of the cells in G0/G1 cell cycle arrest, while high HAS2 expression in stromal cells associates with decreased survival [9, 23]. Decreased hyaluronan synthesis due to knockdown of HAS2 probably explains some of the anti-tumorigenic effects, but HAS2 might harbor some other mechanisms too such as regulating TGFβ-induced epithelial-mesenchymal transition or increasing cancer cell invasion by downregulation of TIMP-1 [10, 24]. Thus HAS2 might utilize some hyaluronan-independent mechanisms that increase cancer cells tumorigenesis.

We also show that HAS1, HAS2 and HYAL2 are associated with WHO grade, suggesting that they have a role in tumor progression. At the moment, the knowledge about the role of hyaluronan synthases and hyaluronidases in diffusely infiltrating astrocytomas is rather limited. Novak et al. (1999) showed that overexpression of HYAL2 in murine astrocytoma leads into faster intracerebral tumor growth and increased tumor vascularization [25]. Hyaluronidases favor cancer progression via production of hyaluronan fragments which enhance cleavage of CD44 and thus induce cell motility. Moreover, hyaluronan fragments promote angiogenesis and increase expression of MMP9 and 13 [26–28]. Overexpression of HAS2 in murine astrocytoma cell lines, unable to produce hyaluronidases, decreased cells capability to form subcutaneous or intracranial tumors, indicating that the activity of both HAS and HYAL are essential for glioma cell invasion [16].

The present data showed that increased HAS2 and HYAL2 intensities are associated with increased cell proliferation. Previously it has been shown that osteopontin-induced HAS2 expression leads to increased hyaluronan synthesis, elevated proliferation and anchorage-independent growth of breast cancer cells [29]. These effects were reversed by inhibition of HAS2 [29]. Similar results have been published with prostate tumor cells [30]. Simpson and co-workers have shown that co-expression of HYAL1 and HAS2 is the most favorable combination for tumor formation in vivo*,* and in vitro overexpression of HAS2 decreases the growth of tumor cells compared to controls [30]. However, co-expression of HYAL1 and HAS2 restored the growth of tumor cells to the same level as control cells [30]. Thus, the results from our work and others suggest that both increased hyaluronan synthase and hyaluronidase activity are required for tumorigenesis.

Glioma patients with *IDH1* or *IDH2* mutation have a significantly better prognosis than patients with wild-type *IDH* [31, 32]. Interestingly, our results indicated that low HAS2 immunostaining intensity associates significantly with *IDH1* mutation. Tumors with *IDH1*-mutation showed mostly weak HAS2 immunostaining intensity compared to wild-type *IDH* tumors which were most often moderately stained for HAS2. Similar association has not been reported before.

One of the hallmarks of grade IV glioblastomas is increased vascularity and many novel promising therapies are antiangiogenic [33]. Low-molecular weight hyaluronan induces angiogenesis facilitating tumor progression [27, 34–36]. Endothelial cell tube formation is dependent on hyaluronan and its receptors, like CD44 and receptor for HA-mediated cell motility (RHAMM) [34, 36]. Furthermore, overexpression of HYAL1 also increases angiogenesis [37]. In our material, expression of CD44 was localized around perivascular areas in tumor tissues. Similar findings have been reported previously with CD44 in astrocytomas where CD44 deficiency led to decreased angiogenesis in mouse model [14, 38]. Angiogenesis might be one way by which hyaluronan and CD44 induce aggressive behavior in diffusely infiltrating astrocytomas.

## Conclusions

The present work demonstrates novel data about changes in hyaluronan metabolism in diffusely infiltrating astrocytomas. We show that HAS1, HAS2 and HYAL2 associate with tumor grade (WHO II-IV), and HAS2 is a negative prognostic factor in diffusely infiltrating astrocytomas. The worsening of outcome can be linked to altered hyaluronan metabolism in tumor microenvironment. Moreover, our results show that there is an association between low HAS2 immunostaining intensity and *IDH1* –mutation, which is a known positive prognostic factor in diffusely infiltrating astrocytomas.

## Additional files

Additional file 1: **Figure S1.** IDH1-R132H –immunostaining in grade II (a) and grade IV tumors (b). Grade II astrocytomas contain several IDH1-R132H –positive cells, while grade IV tumors, glioblastomas, are mostly negative for IDH1-R132H –immunostaining. Scale bar 50 μm. (TIF 33050 kb)

Additional file 2: **Figure S2.** The specificity of the HAS2 stainings was tested with pre-incubating the HAS2 antibody with peptide used in immunization. In A (grade I subependymal giant cell astrocytoma) and C (grade III astrocytoma) HAS2 immunostaining; brown color represents HAS2 and blue indicates nuclei. In B (grade IV gliosarcoma) and D (the grade III astrocytoma) HAS2 antibody was pretreated with peptide. Scale bar 50 μm. (TIF 6383 kb)

Additional file 3: **Table S1.** *p*-values of the other coefficients used in multivariate analyses. (DOCX 15 kb)

## Abbreviations

BSA: Bovine serum albumin
CISH: Chromogenic in situ hybridisation
EGFR: Epidermal growth factor receptor
HAS1–3: Hyaluronan synthases 1–3
HYAL2: Hyaluronidase 2
IDH1–2: Isocitrate dehydrogenase 1–2
MMP-9 and 13: Matrix metalloproteinase-9 and 13
RHAMM: Receptor for HA-mediated cell motility (RHAMM)
TGFβ: Transforming growth factor beta
TIMP-1: Tissue metalloproteinase inhibitor 1
TMA: Tissue microarray
