# Supplementary figures and images for: Elevated expression of hyaluronan synthase 2 associates with decreased survival in diffusely infiltrating astrocytomas
Source: BMC Cancer. 2018 Jun 18;18:664. doi: 10.1186/s12885-018-4569-1 (PMC6006557; doi:10.1186/s12885-018-4569-1)

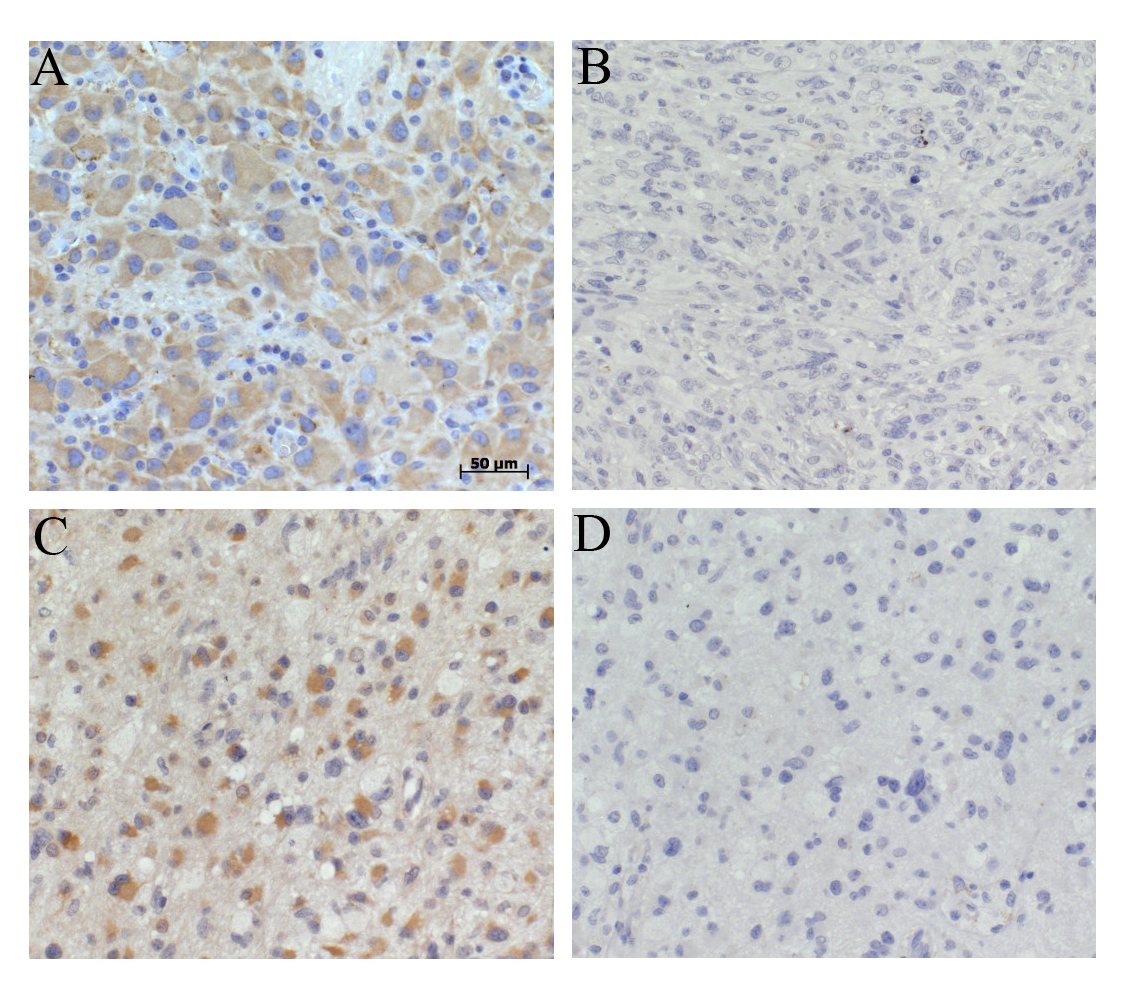

Supplement: Supplementary file 2 — Figure S2. The specificity of the HAS2 stainings was tested with pre-incubating the HAS2 antibody with peptide used in immunization. In A (grade I subependymal giant cell astrocytoma) and C (grade III astrocytoma) HAS2 immunostaining; brown color represents HAS2 and blue indicates nuclei. In B (grade IV gliosarcoma) and D (the grade III astrocytoma) HAS2 antibody was pretreated with peptide. Scale bar 50 μm. (TIF 6383 kb) [file 12885_2018_4569_MOESM2_ESM.tif]
