# Supplementary material for: Elevated expression of hyaluronan synthase 2 associates with decreased survival in diffusely infiltrating astrocytomas
Source: BMC Cancer. 2018 Jun 18;18:664. doi: 10.1186/s12885-018-4569-1 (PMC6006557; doi:10.1186/s12885-018-4569-1)
Supplement: Supplementary file 3 — Table S1. p-values of the other coefficients used in multivariate analyses. (DOCX 15 kb) [file 12885_2018_4569_MOESM3_ESM.docx]

Additional file Table S1. *p*-values of the other coefficients used in multivariate analyses.

| Coefficients | *p*-values |
| --- | --- |
| Grade | 0.354 |
| IDH1 | 0.81 |
| EGFR | 0.201 |
| p53 | 0.674 |
